# Association between short-term air pollution exposure and traumatic intracranial hemorrhage: pilot evidence from Taiwan

**DOI:** 10.3389/fneur.2023.1087767

**Published:** 2023-05-10

**Authors:** Kuo-Hsing Liao, Ta-Chien Chan, Chia-Chieh Wu, Wen-Cheng Huang, Chin-Wang Hsu, Hsiao-Chi Chuang, Bayu Satria Wiratama, Wen-Ta Chiu, Carlos Lam

**Affiliations:** ^1^Department of Neurosurgery, Wan Fang Hospital, Taipei Medical University, Taipei, Taiwan; ^2^Division of Critical Medicine, Department of Emergency and Critical Care Medicine, Wan Fang Hospital, Taipei Medical University, Taipei, Taiwan; ^3^Department of Neurotraumatology and Intensive Care, Taipei Neuroscience Institute, Taipei Medical University, Taipei, Taiwan; ^4^Division of Neurosurgery, Department of Surgery, School of Medicine, College of Medicine, Taipei Medical University, Taipei, Taiwan; ^5^Research Center for Humanities and Social Sciences, Academia Sinica, Taipei, Taiwan; ^6^Institute of Public Health, School of Medicine, National Yang Ming Chiao Tung University, Taipei, Taiwan; ^7^Emergency Department, Wan Fang Hospital, Taipei Medical University, Taipei, Taiwan; ^8^Department of Emergency, School of Medicine, College of Medicine, Taipei Medical University, Taipei, Taiwan; ^9^Emergency Department, Department of Emergency and Critical Care Medicine, Wan Fang Hospital, Taipei Medical University, Taipei, Taiwan; ^10^Center for Education in Medical Simulation, Taipei Medical University, Taipei, Taiwan; ^11^Department of Education and Humanities in Medicine, School of Medicine, College of Medicine, Taipei Medical University, Taipei, Taiwan; ^12^School of Respiratory Therapy, College of Medicine, Taipei Medical University, Taipei, Taiwan; ^13^Division of Pulmonary Medicine, Department of Internal Medicine, Shuang Ho Hospital, Taipei Medical University, Taipei, Taiwan; ^14^Cell Physiology and Molecular Image Research Center, Wan Fang Hospital, Taipei Medical University, Taipei, Taiwan; ^15^Department of Biostatistics, Epidemiology, and Population Health, Faculty of Medicine, Public Health, and Nursing, Universitas Gadjah Mada, Yogyakarta, Indonesia; ^16^Graduate Institute of Injury Prevention and Control, College of Public Health, Taipei Medical University, Taipei, Taiwan; ^17^AHMC Health System, Alhambra, CA, United States

**Keywords:** air pollution, fine particulate matter, geographic information system, road traffic accident, traumatic intracranial hemorrhage, short-term exposure

## Abstract

**Introduction:**

The detrimental effects of air pollution on the brain are well established. However, few studies have examined the effect of air pollution on traumatic brain injury (TBI). This pilot study evaluated the association between short-term air pollution exposure and traumatic intracranial hemorrhage (TIH).

**Methods:**

Hospital data of patients with TBI following road traffic accidents were retrospectively collected from the electronic medical records at five trauma centers in Taiwan between 1 January and 31 December 2017. TIH was employed as an outcome measure. All road accident locations were geocoded, and air quality data were collected from the nearest monitoring stations. Air pollutants were entered into five multivariable models. A sensitivity analysis was performed on patients who are vulnerable to suffering TBI after road accidents, including motorcyclists, bicyclists, and pedestrians.

**Results:**

Among 730 patients with TBI, 327 had TIH. The ages of ≥65 [odds ratio (OR), 3.24; 95% confidence interval (CI), 1.85–5.70], 45–64 (OR, 2.61; 95% CI, 1.64–4.15), and 25–44 (OR, 1.79; 95% CI, 1.13–2.84) years were identified as significant risk factors in the multivariable analysis. In the best-fit multivariable model, exposure to higher concentrations of particulate matter ≤ 2.5 μm in aerodynamic diameter (PM_2.5_) was associated with an elevated TIH risk (OR, 1.50; 95% CI, 1.17–1.94). The concentration of nitrogen oxides (NO_X_) did not increase the risk of TIH (OR, 0.45; 95% CI, 0.32–0.61). After categorizing the air pollution concentration according to quartile, the trend tests in the multivariate model showed that the concentrations of PM_2.5_ and NO_X_ were significant (*p* = 0.017 and *p* < 0.001, respectively). There was a negative borderline significant association between temperature and TIH risk (OR, 0.75; 95% CI, 0.56–1.00, *p* = 0.05). Notably, the single-vehicle crash was a significant risk factor (OR, 2.11; 95% CI, 1.30–3.42) for TIH.

**Discussion:**

High PM_2.5_ concentrations and low temperatures are risk factors for TIH in patients with TBI. High NO_X_ concentrations are associated with a lower TIH risk.

## 1. Introduction

Particulate matter ≤ 10 μm in aerodynamic diameter (PM_10_) was first demonstrated to be strongly correlated with pulmonary disease in the United States in 1989 (1, 2). Air pollutants can harm the cardiovascular system and brain by translocating directly from the olfactory system and lungs through the circulatory system (3–5). Acute exposure to air pollution impairs cognitive function in adults (6), and short-term variations in air pollution are positively correlated with abnormal human behaviors such as aggression and violence (7–9). In addition to their acute neurotoxic effect which may contribute to degenerative brain disorders (10), short-term exposure to air pollution was also reported increasing the risk of hemorrhagic stroke and mortality (11–15). Thus, air pollution may affect the human brain through wider pathways than was previously believed.

An estimated 69 million people worldwide suffer from traumatic brain injury (TBI) each year, and road traffic accidents (RTAs) are the leading cause (16). A Taiwanese study published in 2007 reported that the incidence of TBI following RTAs was 218 per 100,000 population in urban areas and 417 per 100,000 in rural areas (17). Additionally, 67.6% of Taiwan's intracranial hemorrhage were related to RTAs, of which motorcycle-related accidents account for nearly 70% (18). Acute air pollution exposure reduces visibility and irritates drivers' eyes and respiratory tracts. Moreover, studies have reported that air pollution affects drivers' cognitive function and behaviors (6–9). These factors may be responsible for the increased incidence of RTAs linked to acute air pollution exposure (19). A study conducted in the United Kingdom determined a positive correlation between air quality and the number of RTAs, including the concentration of particulate matter ≤ 2.5 μm in aerodynamic diameter (PM_2.5_), nitrogen dioxide (NO_2_), sulfur dioxide (SO_2_), and air quality index (AQI). The author concluded that a higher level of pollutants would likely increase car crashes through its negative effect on safe driving performance (19). An observational study in China reported that short-term exposure to PM_2.5_ and PM_10_ was associated with RTA risk, possibly because of its negative physiological, psychological, and cognitive effects (20). Recently a Taiwanese study reported that the risk of severe road traffic injuries (RTIs) was positively related to acute exposure to poor air quality, including hourly AQI and PM_2.5_ concentrations (21). These investigations explored the association between short-term exposure to air pollution and RTAs and related injury; however, they did not examine the association between air pollutants and the injury severity of a specific human organ (e.g., TBI).

The main sources of ambient air pollution in Taiwan derive from three sources: transport from other countries, local manufactures and construction sites, and transportation industry. Based on estimates from the Taiwan Emission Data System (22), industrial sources account for an average of 27.5% of domestic pollution. Additionally, industrial sources contribute 15, 26, and 40% to the total national emissions of PM_10_, PM_2.5_, and nitrogen oxides (NO_X_), respectively. According to the 2021 statistical report issued by the Ministry of Transportation and Communications (MOTC), the vast number of motorcycles (exceeding 14 million) in Taiwan, mainly light motorcycles with cylinder capacities between 50 and 250 cc, accounts for a considerable share of ambient air pollution (23). Given that acute exposure of air pollution may exert adverse effects on human cognitive function and behavior and impair the visibility of the road users, potentially compromising road traffic safety (6–9), we postulated that acute effect of air pollution would increase the injury severity of RTAs. Therefore, we conducted a pilot study to investigate the association between short-term air pollution exposure and the risk of traumatic intracranial hemorrhage (TIH) attributable to RTIs among patients with TBI. To the best of our knowledge, no other study has examined the association between short-term exposure of air pollutants and TIH. To explain the study results, we applied both human behavior theory and probed potential mechanisms of brain injury following exposure to air pollutants. The findings serve as a reference for road traffic safety, public health authorities, and researchers of TBI to reduce the risk of TIH in TBI following RTAs.

## 2. Materials and methods

### 2.1. Study design

**Figure 1:**
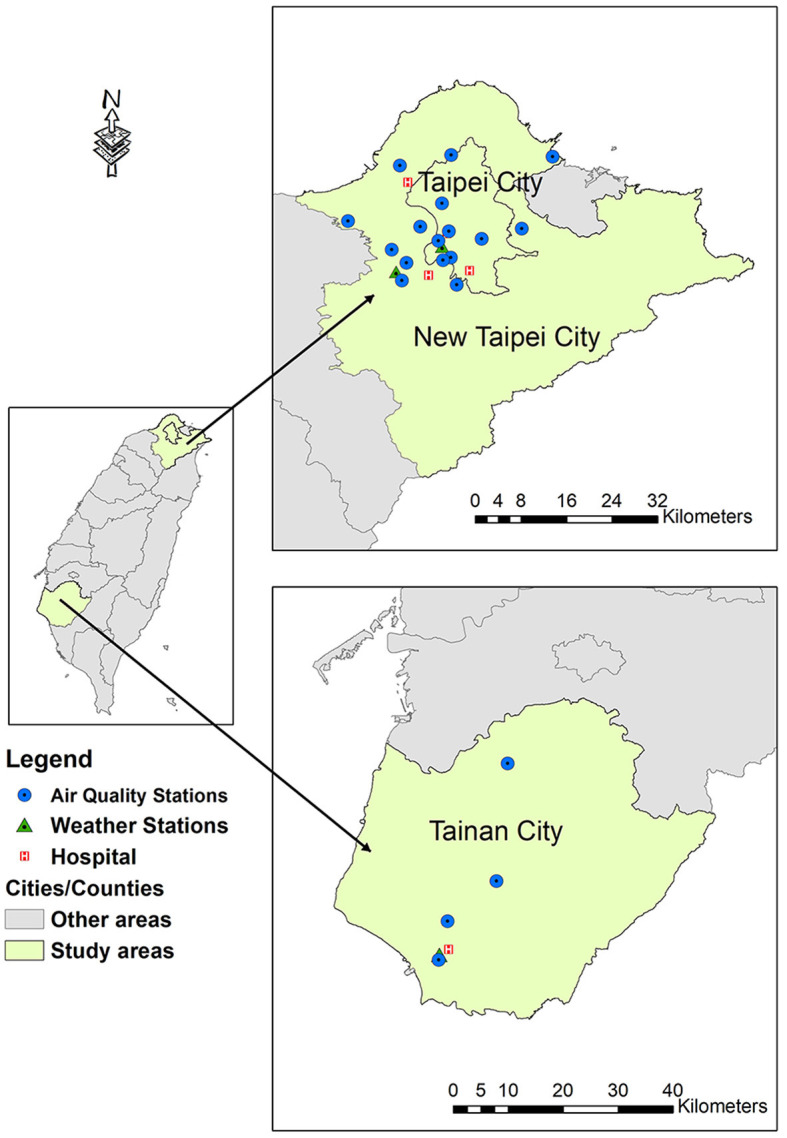
Geographical locations of all participating hospitals and monitoring stations. Modified with permission from Chan et al. (21).

We performed a multicenter cross-sectional study including RTA patients with TBI treated at five trauma centers in Taiwan in 2017: Shuang Ho Hospital and Mackay Memorial Hospital (Tamsui branch) in New Taipei City, Wan Fang Hospital and Mackay Memorial Hospital (Taipei branch) in Taipei City, and National Cheng Kung University Hospital in Tainan City (Figure 1). These university-affiliated teaching hospitals are classified as advanced emergency responsibility hospitals that provide comprehensive care for patients with major trauma. The study protocol was approved by the institutional review boards of participating hospitals (approval nos. 16MMHIS168e, N201510012, and A-ER-105-401). 16MMHIS168e for Mackay Memorial Hospital (Taipei and Tamsui Branch); N201510012 for Taipei Medical University (Shuang Ho Hospital and Wan Fang Hospital); A-ER-105-401 for National Cheng Kung University Hospital.

**Figure 2:**
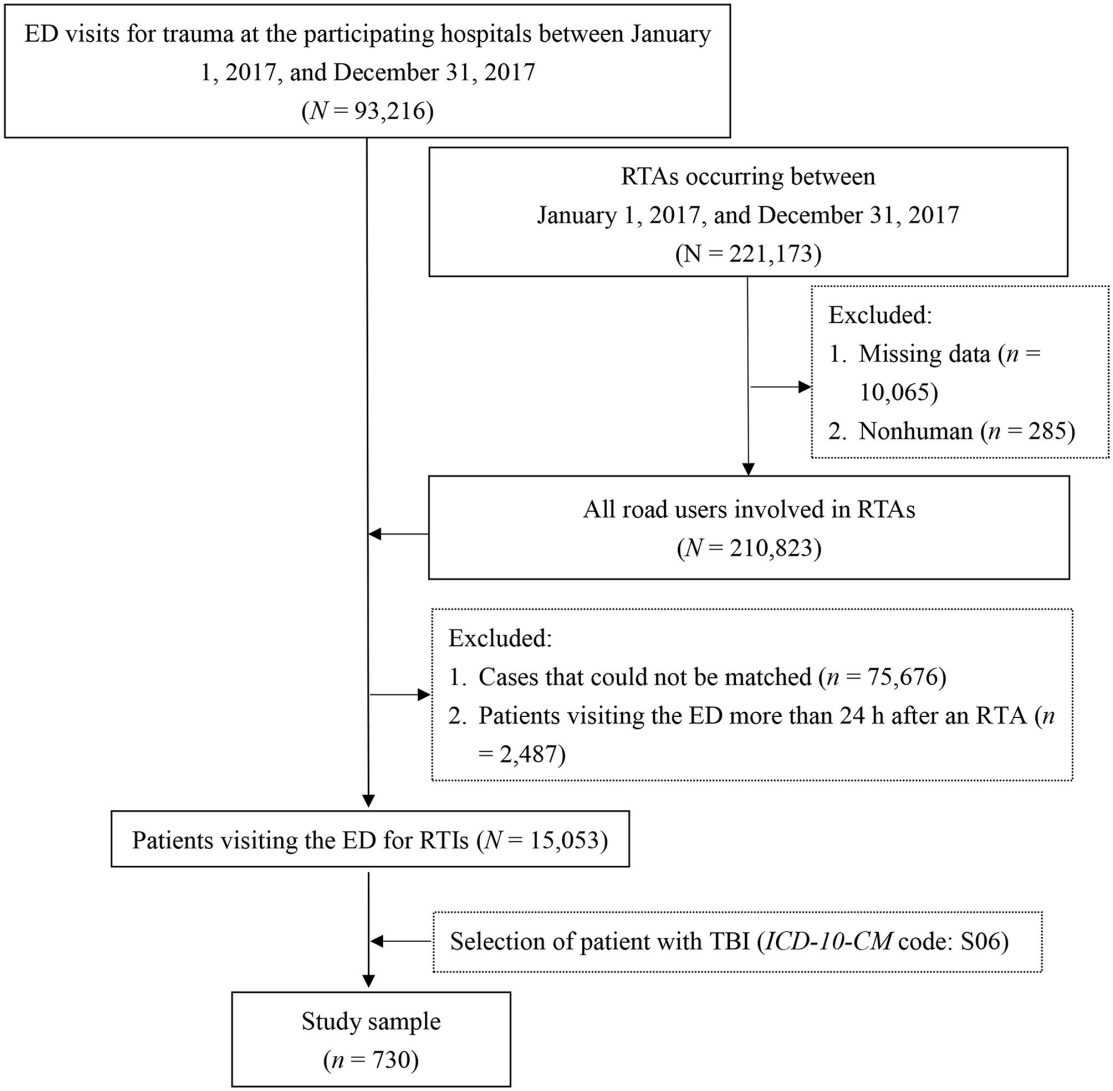
Flowchart of sample selection. ED, emergency department; ICD-10-CM, International Classification of Diseases, Tenth Revision, Clinical Modification; RTAs, road traffic accidents; RTIs, road traffic injuries; TBI, traumatic brain injury.

The hospital data were retrospectively collected from the electronic medical records of trauma patients who had visited the emergency department (ED) of the participating hospitals between January 1, 2017, and December 31, 2017. Patients treated in the ED for RTIs were determined by connecting the hospital data to the police traffic accident data set (PTAD). Thus, information on RTAs in which the patients were involved was obtained. To further secure the causal relationship between the RTAs and the ED visits, we included only patients who presented to the ED within 24 h after the RTA. For the patients who had visited the ED several times after sustaining an RTI, only the record of the first visit was considered. Detailed data processing has been described in published reference (21). In this study, TBI was defined using the S06 codes of the *International Classification of Diseases*, Tenth *Revision, Clinical Modification* (*ICD-10-CM*). The patient selection process is presented in Figure 2.

### 2.2. Measurements

The occurrence of TIH elevates the Head Abbreviated Injury Scale Score (24, 25). Therefore, TIH was employed as an outcome measure indicative of a more severe brain injury, including diffuse brain injury (*ICD-10-CM* code: S06.2), focal brain injury (*ICD-10-CM* code: S06.3), epidural hemorrhage *ICD-10-CM* code: S06.4), traumatic subdural hemorrhage (*ICD-10-CM* code: S06.5), and traumatic subarachnoid hemorrhage (*ICD-10-CM* code: S06.6). The patients in the non-TIH group only had brain concussion (*ICD-10-CM* code: S06.0) without intracranial hemorrhage on brain computed tomography.

Data on the following demographic and environmental factors were retrieved from the PTAD: sex, age (< 25, 25–44, 45–64, and ≥65 years), time of the accident [daytime (06:00–17:59) or evening/nighttime (18:00–05:59)], rush hour status (morning (07:00–09:00), evening (17:00–19:00), and non-rush hour (09:01–16:59 and 19:01–06:59)], crash type (multivehicle, single-vehicle, or pedestrian involvement), scene lighting (daylight, dusk or dawn, or night), and type of road user (vehicle occupant, motorcyclist, bicyclist, or pedestrian).

The air pollutants examined comprised hourly PM_2.5_, PM_10_, O_3_, NO_2_, and NO_X_. We examined the hourly AQI, which was determined according to the maximum subindex values of PM_2.5_, PM_10_, O_3_, SO_2_, carbon monoxide (CO), and NO_2_ (26). The AQI assigns scores of 0 to 500, with a higher score indicating poorer air quality. According to the guidelines of Taiwan's Environmental Protection Administration, the AQI was classified as satisfactory (0–50), moderate (51–100) or unhealthy (>100) (27).

Only one address or location is provided for accidents recorded in the PTAD. Therefore, we used commercial and governmental geocoders, including the Google Maps application programming interfaces (Google Inc., Mountain View, CA, USA) (28) and Taiwan Geospatial One-Stop (29), to geocode the written address or location into geographical coordinates. After the geocodes for all crash sites were ascertained, the corresponding air quality data were collected from the air quality monitoring station nearest to each crash site. Data from 18 stations (15 stations monitoring ambient air quality and 3 stations monitoring traffic air quality) were obtained from the open platform of the Environmental Protection Administration (30).

Data on weather factors, such as hourly mean temperature (in °C) and hourly mean relative humidity (%), were sourced from the Central Weather Bureau (31). The air pollutant concentrations, AQI, temperature, and relative humidity, which were recorded hourly, were highly consistent with the corresponding hour at which the RTAs occurred.

### 2.3. Statistical analysis

Univariate analysis was conducted to evaluate the association between each independent variable and TIH. The Pearson chi-squared test, Cochran–Armitage trend test, and Wilcoxon rank-sum test were applied to categorical, ordered categorical, and continuous variables, respectively. Sex, age, and the independent variables with *p* < 0.2 in the univariate analysis (32) were further included for multivariable analysis.

Because the AQI is an indicator of air quality and includes multiple air pollutants, a separate model was required to circumvent the collinearity problem in the multivariable model. Given the significantly strong correlation between PM_2.5_ and PM_10_ (*r* = 0.861, *p* < 0.001) and between NO_2_ and NO_X_ (*r* = 0.973, *p* < 0.001), these air pollutants were separately entered into five multivariable models (Model 1: AQI; Model 2: PM_2.5_, NO_X_, and O_3_; Model 3: PM_2.5_, NO_2_, and O_3_; Model 4: PM_10_, NO_X_, and O_3_; and Model 5: PM_10_, NO_2_, and O_3_). The final model was generated based on the lowest Akaike Information Criterion value. Odds ratios (ORs) and 95% confidence intervals (Cis) estimated via the multiple logistic regression were used to present the effect of variables on TIH risk.

A trend analysis was performed for ordered categorical variables in the multiple logistic regression analysis. The variance inflation factor (VIF) was used to evaluate collinearity. Variables with VIF scores < 5 were selected. Because vehicle occupants' heads are more protected than those of vulnerable road users, we performed a sensitivity analysis by selecting only motorcyclists, bicyclists, and pedestrians.

A two-sided *p* < 0.05 was considered significant. All statistical analyses were performed using SAS software, Version 9.4 of the SAS System for Unix (SAS Institute Inc., Cary, NC, USA).

## 3. Results

Among the 15,053 patients treated in the five participating hospitals' EDs for RTIs, 730 patients (mean age: 44 years; 52.9% men) had TBI. Among these 730 patients, 327 (44.8%) sustained TIH. The TIH rates among vehicle occupants, motorcyclists, bicyclists, and pedestrians were 29.4, 42.4, 57.4, and 56.5%, respectively.

The results of univariate analysis are presented in Table 1. Significant differences in age; crash type; type of road user; and O_3_, NO_2_, and NO_X_ concentrations were noted.

**Table 1:** Results of univariate analysis between patients with and without TIH.

**Variables**	**Non-TIH (*****n*** = **403)**		**TIH (*****n*** = **327)**		***p*-value**
Sex, no. (%)					0.228^a^
Male	205	(53.11)	181	(46.89)	
Female	198	(57.56)	146	(42.44)	
Median age (IQR), years	36	(22)	48	(37)	< 0.001^b^
Age group, no. (%)					< 0.001^c^
< 25	132	(65.67)	69	(34.33)	
25–44	114	(59.07)	79	(40.93)	
45–64	105	(48.84)	110	(51.16)	
>64	52	(42.98)	69	(57.02)	
Time of crash, no. (%)					0.590^a^
Daytime (06:00–17:59)	247	(56.01)	194	(43.99)	
Evening/nighttime (18:00–05:59)	156	(53.98)	133	(46.02)	
Rush hour, no. (%)					0.652^a^
Morning (07:00–09:00)	51	(56.04)	40	(43.96)	
Evening (17:00–19:00)	70	(58.82)	49	(41.18)	
Non-rush hour (09:01–16:59 and 19:01–06:59)	282	(54.23)	238	(45.77)	
Scene lighting, no. (%)					0.713^a^
Daylight	243	(56.12)	190	(43.88)	
Dusk or dawn	10	(47.62)	11	(52.38)	
Night	149	(54.58)	124	(45.42)	
Missing^d^	1	(0.25)	2	(0.61)	
Crash type, no. (%)					< 0.001^a^
Multivehicle	299	(59.92)	200	(40.08)	
Single-vehicle	42	(43.30)	55	(56.70)	
Pedestrian involvement	61	(46.56)	70	(53.44)	
Missing^d^	1	(0.25)	2	(0.61)	
Type of road user, no. (%)					0.001^a^
Vehicle occupant	36	(70.59)	15	(29.41)	
Motorcyclist	294	(57.65)	216	(42.35)	
Bicyclist	23	(42.59)	31	(57.41)	
Pedestrian	50	(43.48)	65	(56.52)	
Median temperature (IQR), °C	25.7	(9.7)	24.8	(8.7)	0.109^b^
Median relative humidity (IQR), %	71	(15)	72	(27)	0.436^b^
Median AQI (IQR)	51	(33.5)	52	(35)	0.315^b^
AQI level, no. (%)					0.188^a^
Satisfactory (0–50)	199	(56.86)	151	(43.14)	
Moderate (51–100)	169	(55.41)	136	(44.59)	
Unhealthy (>100)	28	(44.44)	35	(55.56)	
Missing^d^	7	(1.74)	5	(1.53)	
Median PM_2.5_ (IQR), μg/m^3^	15	(12)	16	(13)	0.475^b^
Missing^d^	11	(2.73)	6	(1.83)	
Median PM_10_ (IQR), μg/m^3^	32	(21)	34	(25)	0.095^b^
Missing^d^	10	(2.48)	8	(2.45)	
Median O_3_ (IQR), ppb	26.9	(13.7)	29.6	(14.2)	0.020^b^
Missing^d^	38	(9.43)	34	(10.40)	
Median NO_2_ (IQR), ppb	16.8	(12.4)	14.6	(11)	0.005^b^
Missing^d^	12	(2.98)	8	(2.45)	
Median NO_X_ (IQR), ppb	22.0	(17.9)	17.0	(14.8)	0.001^b^
Missing^d^	12	(2.98)	8	(2.45)	

^a^The Pearson chi-squared test. ^b^Wilcoxon rank-sum test. ^c^Cochran–Armitage trend test. ^d^Missing values in the table represent frequencies (column percentages). AQI, air quality index; IQR, interquartile range; NO_2_, nitrogen dioxide; NO_X_, nitrogen oxide; O_3_, ozone; PM_2.5_, particulate matter ≤ 2.5 μm in aerodynamic diameter; PM_10_, particulate matter ≤ 10 μm in aerodynamic diameter; ppb, parts per billion; TIH, traumatic intracranial hemorrhage.

We entered the sex and independent variables with *p* < 0.2 in univariate analysis, including age, crash type, type of road user, and temperature, into multiple logistic regression analyses with air pollutants. The results of all multiple air pollutant models are presented in Supplementary Table S1. All multivariable models consistently demonstrated that air pollutants (excepting AQI) was significantly associated with TIH risk. The best-fit model among these five models is presented in Table 2. A significantly increased TIH risk was observed in the male patients (OR, 1.47; 95% CI, 1.04–2.07). Patients aged ≥65 years had a higher TIH risk than did those aged < 25 years (OR, 3.24; 95% CI, 1.85–5.70). Furthermore, the TIH risk was significantly higher among those aged 25–44 years (OR, 1.79; 95% CI, 1.13–2.84) and 45–64 years (OR, 2.61; 95% CI, 1.64–4.15). The single-vehicle crash was a significant risk factor for TIH compared with a multivehicle crash (OR, 2.11; 95% CI, 1.30–3.42). Compared with the motorcyclists, the vehicle occupants were less likely to sustain a TIH (OR, 0.45; 95% CI, 0.22–0.90). Each interquartile range (IQR) increase in PM_2.5_ was associated with a significant increase in the TIH risk (OR, 1.50; 95% CI, 1.17–1.94).

**Table 2:** Results of multivariable analysis of independent variables and TIH.

**Variables**	**Adjusted OR (95% CI)**	***p*-value**	***p* for trend^a^**
Sex			
Female	Reference		
Male	1.47 (1.04–2.07)	0.031	
Age group, years			< 0.001
< 25	Reference		
25–44	1.79 (1.13–2.84)	0.014	
45–64	2.61 (1.64–4.15)	< 0.001	
>64	3.24 (1.85–5.70)	< 0.001	
Crash type			
Multivehicle	Reference		
Single-vehicle	2.11 (1.30–3.42)	0.003	
Pedestrian involvement	1.23 (0.54–2.83)	0.624	
Type of road user			
Motorcyclist	Reference		
Vehicle occupant	0.45 (0.22–0.90)	0.024	
Bicyclist	1.38 (0.73–2.61)	0.315	
Pedestrian	1.64 (0.66–4.07)	0.286	
Temperature (°C, per IQR)	0.75 (0.56–1.00)	0.050	0.009
PM_2.5_ (μg/m^3^, per IQR)	1.50 (1.17–1.94)	0.002	0.017
NO_X_ (ppb, per IQR)	0.45 (0.32–0.61)	< 0.001	< 0.001
O_3_ (ppb, per IQR)	0.86 (0.64–1.15)	0.311	0.319

Hosmer–Lemeshow goodness-of-fit test: p = 0.605. Effect estimate based on interquartile range (IQR) increases in temperature and air pollutant concentration. The IQRs of temperature, PM_2.5_, NO_X_, and O_3_ were 9.2°C, 13 μg/m^3^, 16.4 ppb, and 14.1 ppb, respectively. ^a^The trends of air pollutants and temperature were estimated by treating the quartiles as a continuous variable. CI, confidence interval; NO_X_, nitrogen oxide; O_3_, ozone; OR, odds ratio; PM_2.5_, particulate matter ≤ 2.5 μm in aerodynamic diameter; ppb, parts per billion; TIH, traumatic intracranial hemorrhage.

IQR increments in NO_X_ were not associated with increases in the TIH risk (OR, 0.45; 95% CI, 0.32–0.61). We observed a borderline negative association of temperature with TIH (OR, 0.75; 95% CI, 0.56–1.00; *p* = 0.05). Overall, significant *p*-values for the positive trends of TIH were found among the following risk factors: age (*p* < 0.001) and PM_2.5_ (*p* = 0.017). For temperature (*p* = 0.009) and NO_X_ (*p* < 0.001), a negative trend was detected.

The results of sensitivity analysis (Table 3) for the association between air pollutants and TIH risk was consistent with the results of multivariable analysis. Exposure to higher PM_2.5_ concentrations was a significant risk factor for TIH (OR: 1.51, 95% CI: 1.16–1.98), whereas NO_X_ was associated with a lower TIH risk (OR, 0.44; 95% CI, 0.32–0.61). Air quality data was tabulated separately to show air quality variations across northern and southern Taiwan (Supplementary Table S2).

**Table 3:** Results of sensitivity analysis.

**Variables**	**Adjusted OR (95% CI)**	***p* value**	***p* for trend^a^**
Sex			
Female	Reference		
Male	1.46 (1.02–2.09)	0.041	
Age group, years			< 0.001
< 25	Reference		
25–44	1.79 (1.11–2.90)	0.017	
45–64	2.91 (1.80–4.68)	< 0.001	
>64	3.42 (1.91–6.10)	< 0.001	
Crash type			
Multivehicle	Reference		
Single-vehicle	1.86 (1.12–3.07)	0.016	
Pedestrian involvement	1.08 (0.44–2.68)	0.866	
Type of road user			
Motorcyclist	Reference		
Bicyclist	1.35 (0.71–2.55)	0.357	
Pedestrian	1.77 (0.67–4.69)	0.252	
Temperature (°C, per IQR)	0.74 (0.55–1.00)	0.050	0.029
PM_2.5_ (μg/m^3^, per IQR)	1.51 (1.16–1.98)	0.003	0.023
NO_X_ (ppb, per IQR)	0.44 (0.32–0.61)	< 0.001	< 0.001
O_3_ (ppb, per IQR)	0.86 (0.63–1.17)	0.330	0.382

Hosmer–Lemeshow goodness-of-fit test: p = 0.954. Effect estimate based on interquartile range (IQR) increases in temperature and air pollutant concentration. The IQRs of temperature, PM_2.5_, NO_X_, and O_3_ were 9.2°C, 13 μg/m^3^, 16.31 ppb, and 14.2 ppb, respectively. ^a^The trends of air pollutants and temperature were estimated by treating the quartiles as a continuous variable. CI, confidence interval; NO_X_, nitrogen oxide; O_3_, ozone; OR, odds ratio; PM_2.5_, particulate matter ≤ 2.5 μm in aerodynamic diameter; ppb, parts per billion.

## 4. Discussion

The present study is the first including a sufficient sample size to report an association between short-term air pollution exposure and TIH in patients with TBI following RTAs. The consistency of the sensitivity analysis results for this association confirms the reliability of our results. To the best of our knowledge, this is the first study to explore the association between short-term air pollution exposure and TIH risk. The high accuracy of spatiotemporal information collected herein promoted the understanding of this association. The time between environmental exposure and the occurrence of an RTA is often short, and the time at which RTAs occur is correlated with various risk factors. In view of such complexities, we employed the precise geographical coordinates of a crash to locate the nearest weather or air quality monitoring station. Relevant investigations have focused mostly on RTA occurrence instead of the severity of injury arising therefrom. Overall, the findings enrich the literature on the connections among air pollution, RTAs, and TIH.

We identified the PM_2.5_ concentration, NO_X_ concentration, age, and temperature as risk factors for TIH following an RTA. We observed a positive dose–response relationship between PM_2.5_ and TIH risk and a negative dose–response relationship between NO_X_ concentration and TIH risk (Supplementary Figure S1).

Short-term air pollution exposure impairs road users' cognitive function and behaviors, increasing the risk of RTAs (19, 20). The aggressive and impulsive behaviors caused by cognitive impairment might result in more severe RTIs, leading to a higher risk of TIH. Local inflammation, endothelial dysfunction, and vasoconstriction in the brain have been implicated as the principal mechanisms through which hemorrhagic stroke is linked to air pollution (33–35). To explain the observed association of short-term air pollution exposure with TIH risk, we further investigated the potential pathway in the central nervous system.

Herein, a dose–response relationship between the PM_2.5_ concentration and TIH risk was noted; exposure to higher PM concentrations was positively associated with TIH risk in individuals sustaining TBI. We proposed three possible mechanisms to explain the short exposure to air pollutants associated with TIH. First, short-term (about 2 h) exposure to air pollutants such as diesel exhaust and higher PM_2.5_ levels (300 μg/m^3^) causes an immediate pollution-attributable decrease in default mode network functional connectivity within the brain (36) while also impairing the cognitive functions and abnormal behaviors such as aggression and violence (6–9, 19, 20). This short-term cognitive dysfunction increases the incidence of RTAs and TIH. Second, short-term exposure to higher PM_2.5_ causes vasoconstriction and a higher risk of hypertension. Experimental studies indicated that exposure to higher levels of PM_2.5_ increases intraaortic levels of Rho-associated kinase (ROCK), which regulates the calcium ion channel of smooth vascular muscle cells (37–39), activating the angiotensin II system and resulting in vasoconstriction and hypertension (40). Another meta-analysis that analyzed 100 articles demonstrated a significant association between hypertension and exposure to PM_10_, PM_2.5_, SO_2_, and NO_2_ levels and a similar association between long-term exposure to PM_2.5_ and hypertension (41). Third, higher PM_2.5_ levels transactivate endothelins and epidermal growth factor receptor (EGFR), which causes proliferation and the subsequent inflammation and dysfunction of the vessel walls of both systemic and intracranial vessels (42–44). Based on the possible mechanisms and our clinical results, exposure to higher PM_2.5_ levels may constitute a higher risk of TIH in RTAs.

NO_X_, which includes nitric oxide (NO) and NO_2_, are commonly found in polluted air. Higher concentrations of NO_X_ were associated with a lower risk of THI in a dose-dependent manner. Inhaled nitric oxide (iNO) has clinically shown a protective effect on central nervous system (45, 46). The extra-pulmonary effect of iNO is considered through the circulating cells exposed to NO in the lungs and blood-borne NO derivatives, such as *S*-nitroso proteins (47–49). The downregulation of lung-derived cytokines and free-radicals production by iNO may also reduce the brain injury (50). The rise in NO levels improves vascular tone and blood flow through the activation of soluble guanylate cyclase in blood vessel walls (51). Moreover, NO counterbalances the vasoconstrictive effect of angiotensin II (52, 53). These mechanisms result in vessel dilation and antihypertension in intracranial vessels, which may reduce the risk of TIH (54).

Traffic congestion during rush hour increases NOx emissions and reduces the severity of RTI because of the decreased vehicle speed. However, our univariable analysis did not show a significant difference in rush hour (*P* = 0.652). The urban and rural samples were then analyzed separately, and again no significant difference was found in rush hour (referred to Supplementary Table S3). Additionally, NO_2_ (NO_X_) is categorized as a secondary pollutant and photochemical oxidant (55). Since we linked the RTA time with the corresponding hourly concentration of air pollutants, the effect of hourly NOx on health outcomes may be delayed (56); therefore, NO_X_ concentration and TIH risk may have a lagged effect. Future investigations should obtain specific data on serum NO_X_ concentrations or fractional exhaled nitric oxide to evaluate the association between atmospheric NO_X_ exposure and serum NO_X_ levels.

Temperature considerably contributes to TIH risk. Low and high TIH risk were observed during warm and cold weather, respectively. When a person rides a motorcycle at 50 km/h in 14°C weather, the rider's apparent temperature is 5°C (57). When a collision involving a rider and pedestrian occurs, the stimulus of pain triggers the sympathetic nervous system and further constricts intracerebral arteries, aggravating the injury to intracerebral vessels and increasing the risk of a TIH. Colder temperatures cause vasoconstriction in the brain and elevate the pressure in intracerebral arteries (58, 59). This explains why the risk of TIH following RTAs is higher in colder weather.

Regarding age as a risk factor for TIH, those aged ≥65 years were more vulnerable to TIH. This is because compared to younger adults, older adults have poorer physical coordination, visual acuity, and muscle strength (sarcopenia in some cases) (60, 61), which is linked to poorer physical function overall in the face of unexpected events such as RTAs. The crash types and road user types were previously reported to correlate with the severity of TBI following RTAs (62–64). Therefore, these two variables were included for adjustment in the multivariable model to diminish their potentially confounding effects and show significant associations with the risk of TIH.

Our multicentric approach strengthened the generalizability of the findings. Our sample was retrieved from five hospitals located in three major cities. These five participating hospitals are all accredited as advanced emergency responsibility hospitals, having capacity to treat patients with severe trauma. Although differences exist between north and south Taiwan in medical resources and urbanization, the five participating hospitals are all in the metropolitan area, resulting in fewer differences. In addition, all hospitals in Taiwan are contracted with the National Health Insurance, which is the single payment system covering 99% of the population. Since the distance between north and south Taiwan is not large, most residents share a similar cultural background and social environment. Therefore, the study's results can be extrapolated to most cities and counties in Taiwan. However, caution must be taken in interpreting the results.

### 4.1. Limitations

This study has several limitations. First, data on preexisting chronic conditions, which may affect the risk of TIH, were not available for analysis. Therefore, we used the patients' age as a proxy. Second, the acquisition of clinical data from hospitals was challenging. Because of our limited resources, we only invited five level-I trauma centers in the metropolitan area to participate in this study. Therefore, sampling bias may exist. Further studies should use a population-based dataset such as the National Health Insurance Research Database to yield more concrete results. Third, the current study only collected short-term air pollution data from the monitoring station nearest to the crash site, which caused data limitations in evaluating long-term exposure. Long-term exposure to air pollution may affect TIH risk. However, the database used in this study did not include patients' residential areas, modes of daily transportation, and other long-term exposure data. Considering the bias created by the crash site data's surrogating long-term data, utilizing a dataset with detailed information on long-term air pollution exposure is crucial for exploring this issue. Fourth, we did not collect data on the level or type of air pollution to which the patients were exposed during the RTAs. These unmeasured factors should be investigated in the future. Finally, gridded data on air pollution would be required to more accurately understand the association between air pollution and different health outcomes.

## 5. Conclusions

Short-term air pollution was associated with TIH risk among the patients with TBI following RTAs. Higher PM_2.5_ concentrations and colder temperatures were associated with an increase in the TIH risk. By contrast, higher NO_X_ concentrations did not increase the risk of TIH. Air pollutants and temperature were associated with TIH risk in a dose-dependent manner. Additional studies with a larger sample size are required to examine the effect of air pollution on patients with TBI as well as the mechanism underlying this effect. The findings can be employed in lowering the risk of TIH following RTIs; furthermore, they can be incorporated into a TBI treatment modality.

## Data availability statement

The raw data supporting the conclusions of this article will be made available by the authors, without undue reservation.

## Ethics statement

The studies involving human participants were reviewed and approved by the Institutional Review Boards of participating hospitals (nos: 16MMHIS168e, N201510012, and A-ER-105-401). N201510012 for Taipei Medical University (Shuang Ho Hospital and Wan Fang Hospital); 16MMHIS168e for Mackay Memorial Hospital (Taipei and Tamsui Branch); A-ER-105-401 for National Cheng Kung University Hospital. Written informed consent for participation was not provided by the participants' legal guardians/next of kin because: the retrospective nature of the study and the high number of patients. This study did not alter the treatment or the life of the patients.

## Author contributions

K-HL and CL contributed to the study concept and design and wrote the draft manuscript. C-CW, W-TC, and CL participated in data acquisition. T-CC, C-CW, W-CH, C-WH, H-CC, BSW, and W-TC contributed to the data analysis and interpretation. All authors critically revised the manuscript for important intellectual content and approved the final version.

## Supplementary material

Supplementary Table S1 Results of all multiple air pollutant models.

Supplementary Table S2 The distribution of air quality in the study areas.

Supplementary Table S3 Comparison of the rush hour between patients with or without TIH in urban and rural samples.

Supplementary Figure S1 The dose-response relationship showing the change of TIH risk related to short-term air pollutants concentrations of **(A)** PM_2.5_ and **(B)** NO_X_.
