# Supplementary material for: Association between short-term air pollution exposure and traumatic intracranial hemorrhage: pilot evidence from Taiwan
Source: Front Neurol. 2023 May 10;14:1087767. doi: 10.3389/fneur.2023.1087767 (PMC10208221; doi:10.3389/fneur.2023.1087767)
Supplement: Supplementary Table S1 — Results of all multiple air pollutant models. [file Table_1.pdf]

Table S1. Results of all multiple air pollutant models.

Variables	Model 1 (AQI)			Model 2 ($PM_{2.5}$, NO_X, O_3)			Model 3 ($PM_{2.5}$, NO_2, O_3)			Model 4 (PM_{10}, NO_X, O_3)			Model 5 (PM_{10}, NO_2, O_3)		
	**OR**	**95% CI**	***p* for trend**	**OR**	**95% CI**	***p* for trend**	**OR**	**95% CI**	***p* for trend**	**OR**	**95% CI**	***p* for trend**	**OR**	**95% CI**	***p* for trend**
**AQI level** *(Reference: Satisfactory (0–50))*			0.381												
Moderate (51–100)	1.04	(0.75–1.44)													
Unhealthy (>100)	1.38	(0.78–2.44)													
**$PM_{2.5}$ (μg/m^3, per IQR)**				**1.50**	(1.17–1.94)	0.017	**1.50**	(1.16–1.96)	0.017						
**PM_{10} (μg/m^3, per IQR)**										**1.48**	(1.15–1.90)	0.023	**1.48**	(1.15–1.92)	0.021
**NO_X (ppb, per IQR)**				**0.45**	(0.32–0.61)	<0.001				**0.48**	(0.35–0.65)	<0.001			
**NO_2 (ppb, per IQR)**							**0.46**	(0.33–0.65)	<0.001				**0.50**	(0.36–0.69)	<0.001
**O_3 (ppb, per IQR)**				0.86	(0.64–1.15)	0.319	0.95	(0.71–1.26)	0.667	0.89	(0.67–1.19)	0.407	0.98	(0.74–1.29)	0.776
**Temperature (°C, per IQR)**	0.81	(0.63–1.04)	0.018	0.75	(0.56–1.00)	0.009	0.75	(0.56–1.01)	0.008	0.75	(0.56–1.01)	0.020	0.76	(0.57–1.01)	0.018
**Sex** *(Reference: Female)*															
Male	**1.43**	(1.04–1.97)		**1.47**	(1.04–2.07)		**1.46**	(1.03–2.05)		**1.42**	(1.00–2.00)		**1.41**	(1.00–1.98)	
**Age group, years** *(Reference: < 25)*			<0.001			<0.001			<0.001			<0.001			<0.001
25–44	1.49	(0.97–2.27)		**1.79**	(1.13–2.84)		**1.73**	(1.09–2.73)		**1.74**	(1.10–2.75)		**1.68**	(1.07–2.66)	
45–64	**2.08**	(1.36–3.18)		**2.61**	(1.64–4.15)		**2.57**	(1.62–4.09)		**2.50**	(1.58–3.97)		**2.48**	(1.56–3.93)	
> 64	**2.35**	(1.41–3.94)		**3.24**	(1.85–5.70)		**3.17**	(1.81–5.57)		**3.11**	(1.77–5.46)		**3.04**	(1.73–5.32)	
**Crash type** *(Reference: Multivehicle)*															
Single-vehicle	**2.11**	(1.34–3.33)		**2.11**	(1.30–3.42)		**2.11**	(1.30–3.41)		**2.09**	(1.29–3.40)		**2.09**	(1.29–3.39)	
Pedestrian involvement	0.93	(0.42–2.07)		1.23	(0.54–2.83)		1.29	(0.57–2.97)		1.38	(0.59–3.20)		1.44	(0.62–3.35)	
**Type of road user** *(Reference: Motorcyclist)*															
Bicyclist	1.61	(0.88–2.93)		1.38	(0.73–2.61)		1.38	(0.73–2.59)		1.44	(0.76–2.75)		1.43	(0.75–2.73)	
Pedestrian	1.88	(0.80–4.43)		1.64	(0.66–4.07)		1.54	(0.62–3.80)		1.32	(0.53–3.30)		1.24	(0.50–3.09)	
Vehicle occupant	**0.51**	(0.27–0.98)		**0.45**	(0.22–0.90)		**0.46**	(0.23–0.91)		**0.44**	(0.22–0.89)		**0.45**	(0.22–0.90)	

Model fit statistics					
AIC	959.755	834.189	841.069	837.744	843.979
-2 Log likelihood	933.755	806.189	813.069	809.744	815.979
Hosmer–Lemeshow goodness-of-fit test	$\chi^2 = 8.5104$<br>$p = 0.201$	$\chi^2 = 6.3759$<br>$p = 0.605$	$\chi^2 = 9.9248$<br>$p = 0.270$	$\chi^2 = 8.5104$<br>$p = 0.385$	$\chi^2 = 7.4234$<br>$p = 0.492$

Effect estimate based on interquartile range (IQR) increases in temperature and air pollutant concentration. The IQRs of temperature, $PM_{2.5}$, PM_{10}, NO_X, NO_2, and O_3 were 9.2℃, 13 μg/m^3, 23 μg/m^3, 16.4 ppb, 12.16 ppb, and 14.1 ppb, respectively.

The trends of air pollutants and temperature were estimated by treating the quartiles as a continuous variable.

AIC, Akaike Information Criterion; AQI, air quality index; CI, confidence interval; IQR, interquartile range; NO_2, nitrogen dioxide; NO_X, nitrogen oxide; O_3, ozone; OR, odds ratio; $PM_{2.5}$, particulate matter ≤2.5 μm in aerodynamic diameter; PM_{10}, particulate matter ≤10 μm in aerodynamic diameter; ppb, parts per billion.
