# Supplementary material for: Association between short-term air pollution exposure and traumatic intracranial hemorrhage: pilot evidence from Taiwan
Source: Front Neurol. 2023 May 10;14:1087767. doi: 10.3389/fneur.2023.1087767 (PMC10208221; doi:10.3389/fneur.2023.1087767)
Supplement: Supplementary Table S2 — The distribution of air quality in the study areas. [file Table_2.pdf]

Table S2: The distribution of air quality in the study areas.

Air pollutants	Taipei City (n = 185)	New Taipei City (n = 461)	Tainan City (n = 84)
AQI level, no. (%)			
Satisfactory (0–50)	84 (46.4)	242 (53.2)	24 (29.3)
Moderate (51–100)	89 (49.2)	189 (41.5)	27 (32.9)
Unhealthy (>100)	8 (4.4)	24 (5.3)	31 (37.8)
Missing	4 (2.2)	6 (1.3)	2 (2.4)
Median $PM_{2.5}$ (IQR), μg/m^3	15 (11)	14 (12)	24.5 (25)
Median PM_{10} (IQR), μg/m^3	33 (22)	32 (22)	49 (42)
Median O_3 (IQR), ppb	27.2 (13.7)	28.2 (14.0)	28.6 (13.6)
Median NO_2 (IQR), ppb	20.9 (10.5)	14.9 (10.3)	13.9 (8.0)
Median NO_X (IQR), ppb	29.1 (18.3)	19 (16.0)	15.8 (8.6)

AQI, air quality index; IQR, interquartile range; NO_2, nitrogen dioxide; NO_X, nitrogen oxide; O_3, ozone; $PM_{2.5}$, particulate matter ≤2.5 μm in aerodynamic diameter; PM_{10}, particulate matter ≤10 μm in aerodynamic diameter; ppb, parts per billion.
