# Supplementary material for: Association between short-term air pollution exposure and traumatic intracranial hemorrhage: pilot evidence from Taiwan
Source: Front Neurol. 2023 May 10;14:1087767. doi: 10.3389/fneur.2023.1087767 (PMC10208221; doi:10.3389/fneur.2023.1087767)
Supplement: Supplementary Table S3 — Comparison of the rush hour between patients with or without TIH in urban and rural samples. [file Table_3.pdf]

Table S3. Comparison of the rush hour between patients with or without TIH in urban and rural samples.

Variables	Non-TIH		TIH		*p* value
	**n**	**%**	**n**	**%**	
Urban samples					
Rush hour					0.814
Morning (07:00–09:00)	48	57.14	36	42.86	
Evening (17:00–19:00)	65	59.09	45	40.91	
Non-rush hour (09:01–16:59 and 19:01–06:59)	274	55.80	217	44.20	
Rural samples					
Rush hour					0.316[a]
Morning (07:00–09:00)	3	42.86	4	57.14	
Evening (17:00–19:00)	5	55.56	4	44.44	
Non-rush hour (09:01–16:59 and 19:01–06:59)	8	27.59	21	72.41	

TIH, traumatic intracranial hemorrhage.
