# Supplementary figures and images for: Association between short-term air pollution exposure and traumatic intracranial hemorrhage: pilot evidence from Taiwan
Source: Front Neurol. 2023 May 10;14:1087767. doi: 10.3389/fneur.2023.1087767 (PMC10208221; doi:10.3389/fneur.2023.1087767)

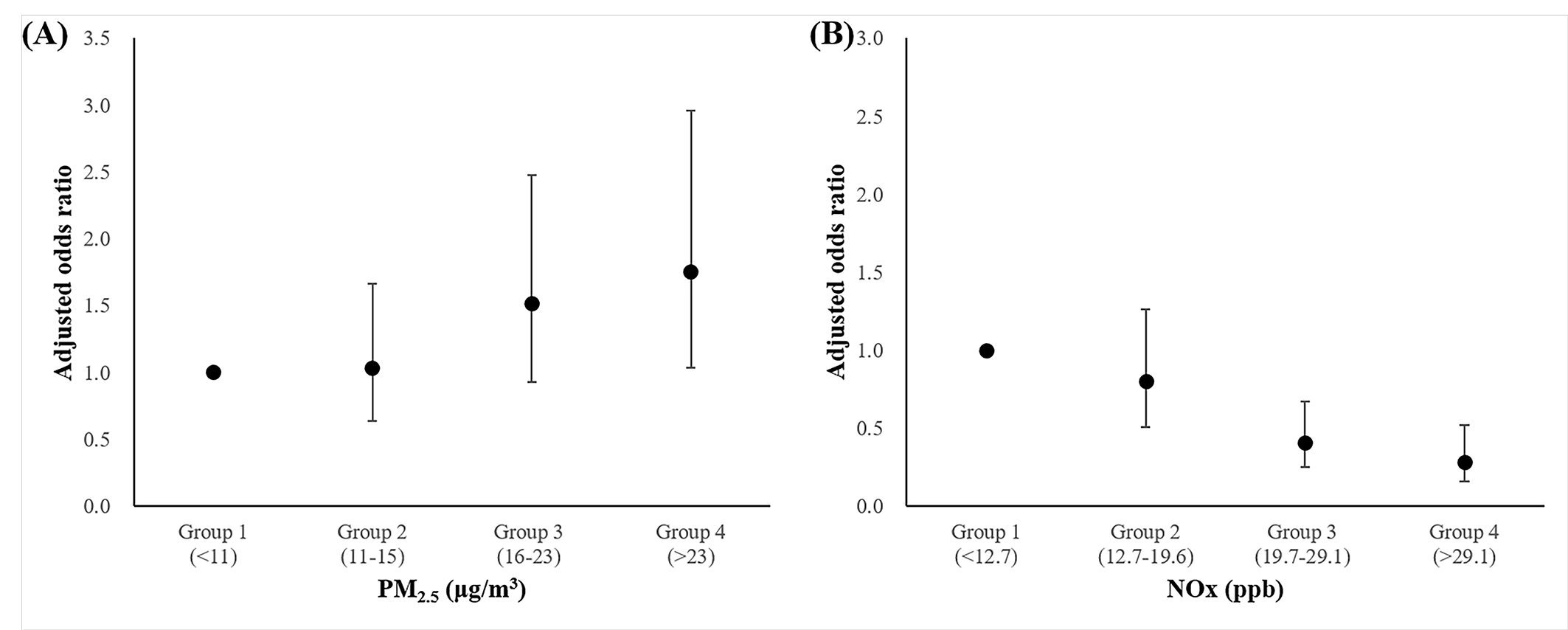

Supplement: Supplementary Figure S1 — The dose-response relationship showing the change of TIH risk related to short-term air pollutants concentrations of (A) PM2.5 and (B) NOX. [file Image_1.TIFF]
